# A Commentary on “Accelerated biological aging and postoperative acute kidney injury in surgical patients”

**DOI:** 10.1097/JS9.0000000000004559

**Published:** 2026-01-12

**Authors:** Changyuan Yang, Jiachuan Xiong

**Affiliations:** a Department of Nephrology, University Medical Center Groningen, University of Groningen, Groningen, The Netherlands; b Department of Nephrology, The Key Laboratory for the Prevention and Treatment of Kidney Disease of Chongqing, Chongqing Clinical Research Center of Kidney and Urology Diseases, Xinqiao Hospital, Army Medical University (Third Military Medical University), Chongqing, China

*Dear Editor,*

We read with great interest the article by Yin *et al*[1], which analyzed a large cohort of 94 006 patients and concluded that accelerated biological aging, as measured by Phenotypic Age Acceleration (PhenoAgeAccel), is an independent risk factor for postoperative acute kidney injury (AKI), AKI severity, and prolonged length of stay, which is highly significant. Notably, the authors reported that this association remained significant even after excluding baseline creatinine from the PhenoAge calculation, suggesting that its predictive value may be driven primarily by non-renal components such as inflammation and immunosenescence. This work highlights the growing relevance of biological aging in perioperative management, particularly its potential utility in predicting and informing surgical patients about their risk of developing AKI postoperatively. However, several methodological and interpretive issues merit careful consideration before the clinical applicability of these findings can be fully established.

First, regarding the biological age metric selection, while PhenoAge was a pioneering tool, it relies heavily on routine clinical biomarkers susceptible to acute, non-aging-related fluctuations[2]. In the present cohort, 37.7% of patients were classified as “biologically older,” yet many PhenoAge components – particularly CRP and glucose – are sensitive to subclinical infection, cancer-related inflammation, or preoperative stress, raising concerns about potential inflation of biological aging estimates. This instability is further reflected in the observation that the association between accelerated biological aging and AKI lost statistical significance in the oldest old (≥80 years). Because different biological aging algorithms vary substantially in their stability under acute physiological stress, a comparison with alternative measures – such as the recently validated GOLD bioage algorithm, which has demonstrated superior predictive performance – would strengthen confidence in the robustness of the observed associations[3].

Second, several features of the cohort and data structure raise concerns regarding potential bias and residual confounding. For example, the reported prevalence of hypertension (3.5%), diabetes (1.6%), and chronic kidney disease (CKD) (0.4%) is strikingly low for a surgical population with a median age of 62 years in China^[4,5]^. Such discrepancies suggest substantial under-coding or incomplete capture of comorbidity data, which may undermine the validity of multivariable adjustment and exaggerate the independence of PhenoAgeAccel. In addition, more than 173 000 patients – nearly twice the size of the final analytic cohort – were excluded because they did not survive or were not followed for at least 7 days postoperatively. As postoperative AKI is defined within a 7-day window, this exclusion strategy likely removes the sickest individuals, creating a healthy survivor bias and potentially attenuating or distorting the true association between biological aging and AKI. In addition, the cohort also included a high proportion of patients with cancer (14.5%), among whom chemotherapy and immunotherapy can significantly influence leukocyte indices and lymphocyte proportions[6]. The calculated biological age may, therefore, partially reflect treatment-related hematological effects rather than the intrinsic aging process. More robust confounding adjustment using propensity score matching, inverse probability weighting, or stratification could help mitigate these concerns[7].

Third, Data handling further complicates interpretation. CRP – a central component of PhenoAge – was missing in 73.53% of participants, but the authors relied on mean imputation. This approach compresses variance, biases the biological age distribution, and may meaningfully alter risk estimates. Although the authors performed a complete-case analysis, this subgroup likely represents a healthier subset and does not address the bias introduced in the primary analysis[8].

Finally, while the study demonstrates the association between preoperative PhenoAgeAccel and AKI incidence, the clinical narrative regarding predictive utility and long-term outcomes remains underdeveloped. For biological age metrics to have meaningful clinical utility in perioperative management, they should demonstrate incremental risk discrimination beyond existing clinical models, adequate calibration across diverse surgical populations, and actionable implications that can guide intervention[9]. A significant unresolved question is the clinical utility of PhenoAgeAccel in differentiating patients with AKI who will experience recovery from those who will progress to acute kidney disease or CKD. Furthermore, the possibility of reverse causation warrants consideration: specifically, whether AKI – as a profound physiological stressor – contributes to accelerated postoperative biological aging[10]. Elucidating these bidirectional relationships will be crucial for understanding long-term outcomes.

In conclusion, the work by Yin *et al*[1] provides an important foundation for integrating biological aging into perioperative risk assessment. Its clinical translation, however, requires more rigorous validation and transparent methodology. Such efforts will strengthen the evidence base for adopting biological age as a meaningful perioperative biomarker.

## Data Availability

No new data were generated or analyzed in this commentary.
